# Supplementary material for: High-throughput screening of the ReFRAME, Pandemic Box, and COVID Box drug repurposing libraries against SARS-CoV-2 nsp15 endoribonuclease to identify small-molecule inhibitors of viral activity
Source: PLoS One. 2021 Apr 22;16(4):e0250019. doi: 10.1371/journal.pone.0250019 (PMC8062000; doi:10.1371/journal.pone.0250019)
Supplement: S1 Fig — A) Zoom-in view of the 6mers peaks from SID spectra at different Exebryl-1 concentration. Significant mass increase was detected as ligand concentration increased, as seen by the shift of all peaks to the right. B) Average ligand per 1mer based on the data from released 1mers (filled circles) and mass shift on the 6mers (open squares) from SID (red) and CID (blue). The total mass shifts on the 6mer was divided by the mass of Exebryl-1 to yield the total number of ligands. Then the number was further divided by 6 to yield the average ligand per 1mer, assuming all ligands are symmetrically distributed within the 6mer. The mass shifts correlate well with the mass shift on 1mers, suggesting Exebryl-1 binds to all 1mers in the complex symmetrically. The systematic higher values calculated from 6mers than from 1mers are likely due to additional nonspecific salt/solvent addition to the 6mers and potential loss of weakly associated exebryl-1. CID consistently showed lower ligand numbers on both 1mers and 6mers, especially at increased ligand concentration. (DOCX) [file pone.0250019.s001.docx]

| **A.** Zoom of the 6mer peaks in the SID spectra  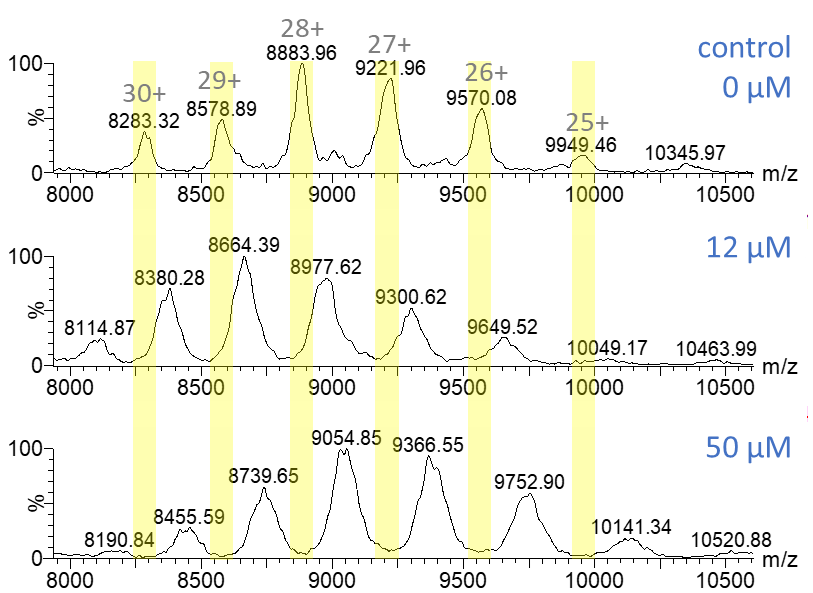 | **B.** Average ligand per 1mer   |
| --- | --- |

**S1 Fig. Exebryl-1 mass addition on the nsp15 hexamer**. **A)** Zoom-in view of the 6mers peaks from SID spectra at different Exebryl-1 concentration. Significant mass increase was detected as ligand concentration increased, as seen by the shift of all peaks to the right. **B)** Average ligand per 1mer based on the data from released 1mers (filled circles) and mass shift on the 6mers (open squares) from SID (red) and CID (blue). The total mass shifts on the 6mer was divided by the mass of Exebryl-1 to yield the total number of ligands. Then the number was further divided by 6 to yield the average ligand per 1mer, assuming all ligands are symmetrically distributed within the 6mer. The mass shifts correlate well with the mass shift on 1mers, suggesting Exebryl-1 binds to all 1mers in the complex symmetrically. The systematic higher values calculated from 6mers than from 1mers are likely due to additional nonspecific salt/solvent addition to the 6mers and potential loss of weakly associated exebryl-1. CID consistently showed lower ligand numbers on both 1mers and 6mers, especially at increased ligand concentration.
