# Supplementary material for: High-throughput screening of the ReFRAME, Pandemic Box, and COVID Box drug repurposing libraries against SARS-CoV-2 nsp15 endoribonuclease to identify small-molecule inhibitors of viral activity
Source: PLoS One. 2021 Apr 22;16(4):e0250019. doi: 10.1371/journal.pone.0250019 (PMC8062000; doi:10.1371/journal.pone.0250019)
Supplement: S2 Fig — A) Full views of 90 V SID for mass isolated nsp15 6mer. B) Zoom view of the released 1mer in the SID spectra. C) Full views of 100 V CID for mass isolated nsp15 6mer. D) Zoom view of the released 1mer in the CID spectra. The top and middle panels in each panel are the spectrum with compounds added (Piroxantrone and MMV1580853, respectively), the bottom panel is control. The same experimental methods used for exebryl-1 binding was applied here. Nsp15 at 5 μM concentration in 100 mM ammonium acetate (pH 7.5) was mixed with 10 μM Mn(II) acetate, and 10 μM compound. The compound was diluted from 10 mM stock in DMSO. For control, pure DMSO was used as stock. No significant amount of piroxantrone or MMV1580853 can be detected in the released 1mers in both SID and CID at 10 μM compound concentration. However, the compounds were released from the 6mer upon activation, as shown by the strong signal at low m/z (412.2 and 529.2 for protonated piroxantrone and MMV1580853, respectively). The data suggest these compounds weakly bind to nsp15. (DOCX) [file pone.0250019.s002.docx]

| **A.** SID of mass-isolated nsp15 6mer  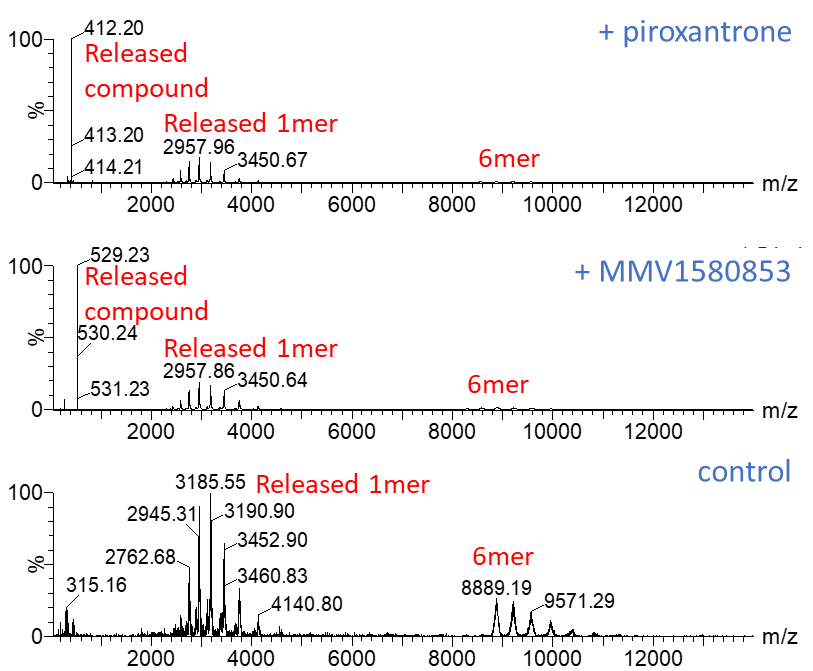 | **B.** Zoom of released 1mer (13+) in SID  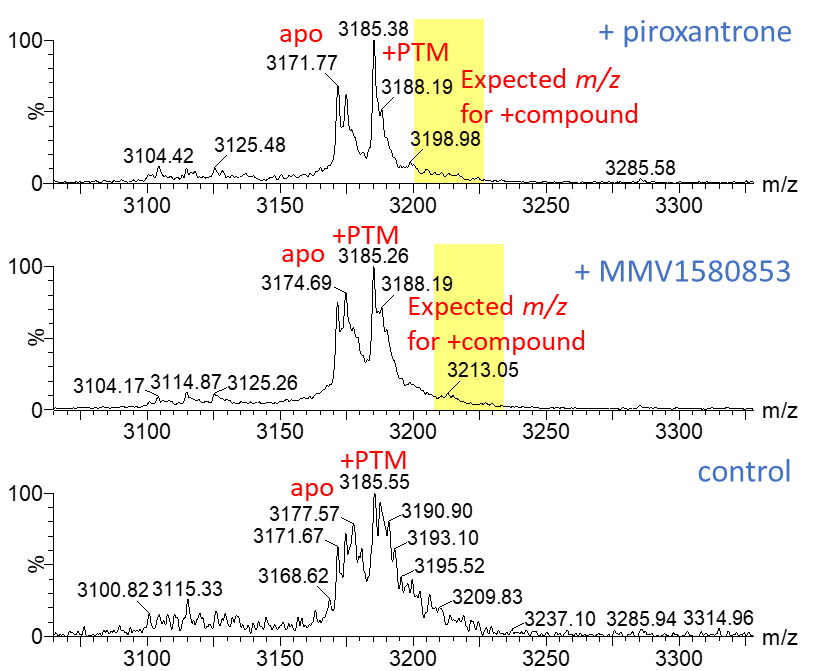 |
| --- | --- |
| **C.** CID of mass-isolated nsp15 6mer  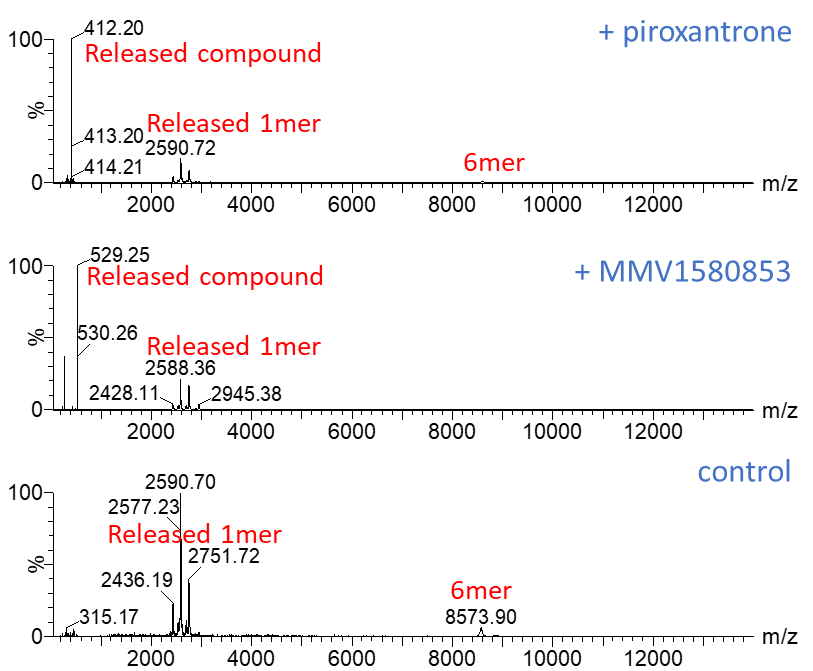 | **D.** Zoom of released 1mer (16+) in CID  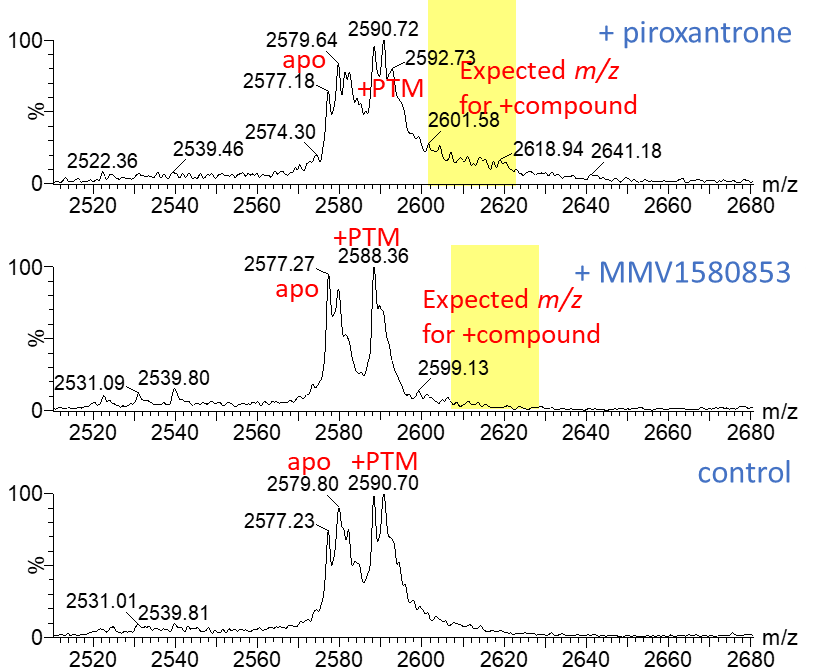 |

**S2 Fig. Native MS analysis of nsp15 binding to Piroxantrone and MMV1580853. A)** Full views of 90 V SID for mass isolated nsp15 6mer. **B)** Zoom view of the released 1mer in the SID spectra. **C)** Full views of 100 V CID for mass isolated nsp15 6mer. **D)** Zoom view of the released 1mer in the CID spectra. The top and middle panels in each panel are the spectrum with compounds added (Piroxantrone and MMV1580853, respectively), the bottom panel is control. The same experimental methods used for exebryl-1 binding was applied here. Nsp15 at 5 µM concentration in 100 mM ammonium acetate (pH 7.5) was mixed with 10 µM Mn(II) acetate, and 10 µM compound. The compound was diluted from 10 mM stock in DMSO. For control, pure DMSO was used as stock. No significant amount of piroxantrone or MMV1580853 can be detected in the released 1mers in both SID and CID at 10 µM compound concentration. However, the compounds were released from the 6mer upon activation, as shown by the strong signal at low *m/z* (412.2 and 529.2 for protonated piroxantrone and MMV1580853, respectively). The data suggest these compounds weakly bind to nsp15.
