# Supplementary material for: High-throughput screening of the ReFRAME, Pandemic Box, and COVID Box drug repurposing libraries against SARS-CoV-2 nsp15 endoribonuclease to identify small-molecule inhibitors of viral activity
Source: PLoS One. 2021 Apr 22;16(4):e0250019. doi: 10.1371/journal.pone.0250019 (PMC8062000; doi:10.1371/journal.pone.0250019)
Supplement: S3 Fig — A) MMV1580853 binding mode 1 in a deep pocket formed between the C-terminal endoU and N-terminal oligomerization domains and oriented within the pore structure of the catalytically active hexamer. B) MMV1580853 binding mode 2 in the endoU catalytic site (relatively less stable by 1.1 kcal/mol, lower binding affinity as compared to mode 1). C) Piroxantrone binding mode 1 within the pore structure. D) Piroxantrone binding mode 2 in the endoU catalytic site (relatively less stable by ~0.6 kcal/mol, lower binding affinity as compared to mode 1). MMV1580853 and Piroxantrone appear to bind on the surface in both binding modes rather than buried within the binding pockets. (DOCX) [file pone.0250019.s003.docx]

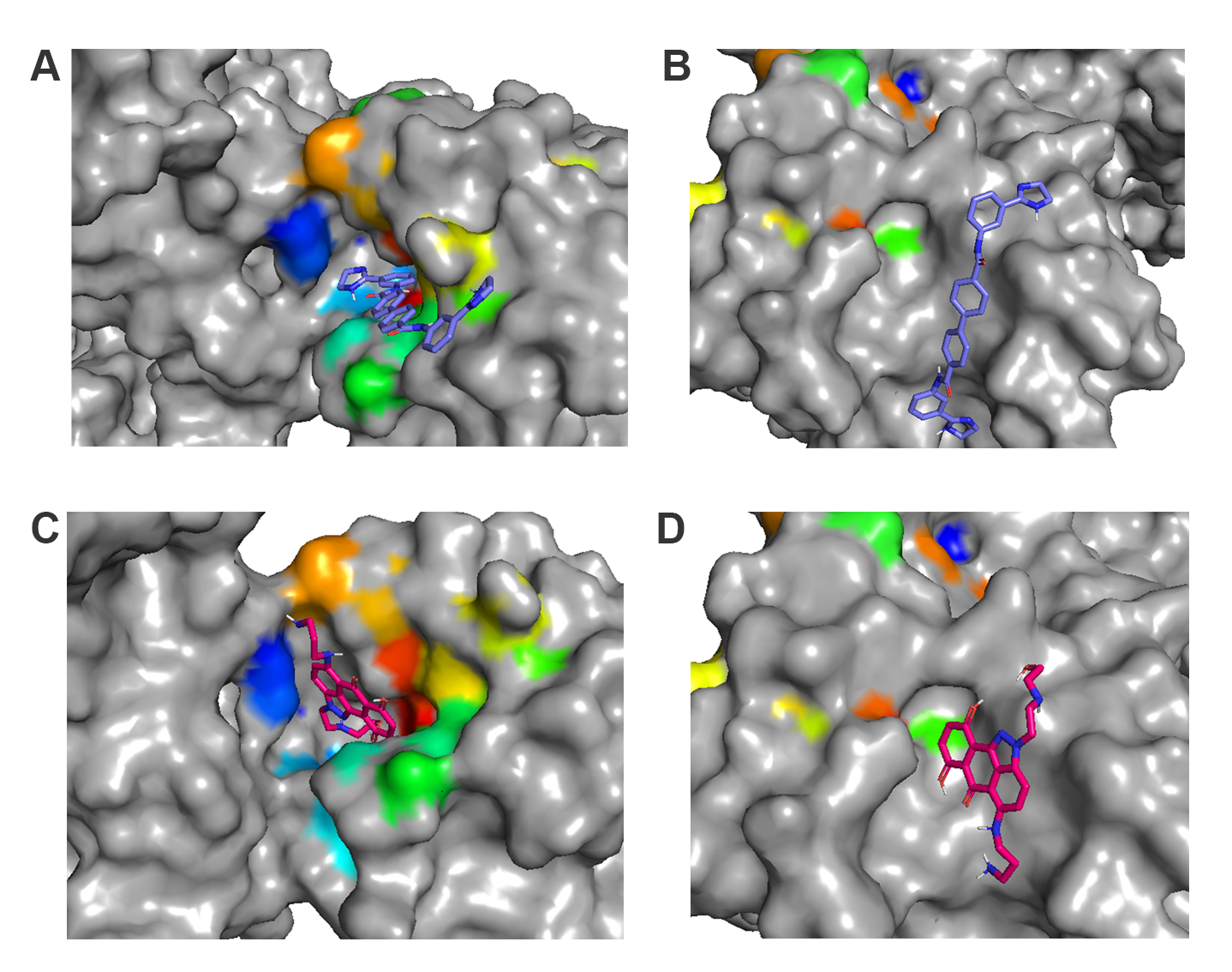


**S3 Fig. Molecular docking of MMV1580853 and Piroxantrone.** A) MMV1580853 binding mode 1 in a deep pocket formed between the C-terminal endoU and N-terminal oligomerization domains and oriented within the pore structure of the catalytically active hexamer. B) MMV1580853 binding mode 2 in the endoU catalytic site (relatively less stable by 1.1 kcal/mol, lower binding affinity as compared to mode 1). C) Piroxantrone binding mode 1 within the pore structure. D) Piroxantrone binding mode 2 in the endoU catalytic site (relatively less stable by ~0.6 kcal/mol, lower binding affinity as compared to mode 1). MMV1580853 and Piroxantrone appear to bind on the surface in both binding modes rather than buried within the binding pockets.
