# Supplementary material for: High-throughput screening of the ReFRAME, Pandemic Box, and COVID Box drug repurposing libraries against SARS-CoV-2 nsp15 endoribonuclease to identify small-molecule inhibitors of viral activity
Source: PLoS One. 2021 Apr 22;16(4):e0250019. doi: 10.1371/journal.pone.0250019 (PMC8062000; doi:10.1371/journal.pone.0250019)
Supplement: S4 Fig — A brief description of the synthesis is as follows: to a suspension of protected benzoic acid (1) (0.64 g, 3.85 mmol) in THF (10.0 mL) was added hydorxybenzotriazole (HOBt, 0.52 g, 3.85 mmol), 1-ethyl-3-(3-dimethylaminopropyl)carbodiimide (EDC.HCl, 0.738 g, 3.85 mmol) and diisopropylethylamine (DIPEA, 1.83 mL, 10.5 mmol) at room temperature and stirred for 30 min. To this now clear solution the protected aniline (2) was added (0.48 g, 2.9 mmol) and the mixture was stirred at room temperature overnight. The solvent and excess reagent were removed under reduced pressure and the residue was purified on silica gel with 10% EtOAc/hexanes to provide 0.56 g (62%) of the intermediate (3) as pinkish solids. To a solution of the intermediate product (3) (0.56 g, 1.69 mmol) in DCM (17 mL) was added BBr3 (0.65 mL, 6.76 mmol) at 0°C, the mixture was allowed to warm to room temperature and stirred overnight. Methanol was added slowly until the suspension became clear. The solvents and excess reagent were removed under reduced pressure and the residue was purified on silica gel with 60% EtOAc/hexanes followed by 10% MeOH/DCM to provide the product with pink color. The material was suspended and stirred in DCM, filtered and washed with DCM to provide the final product as white solids (0.3 g, 68%). (DOCX) [file pone.0250019.s004.docx]

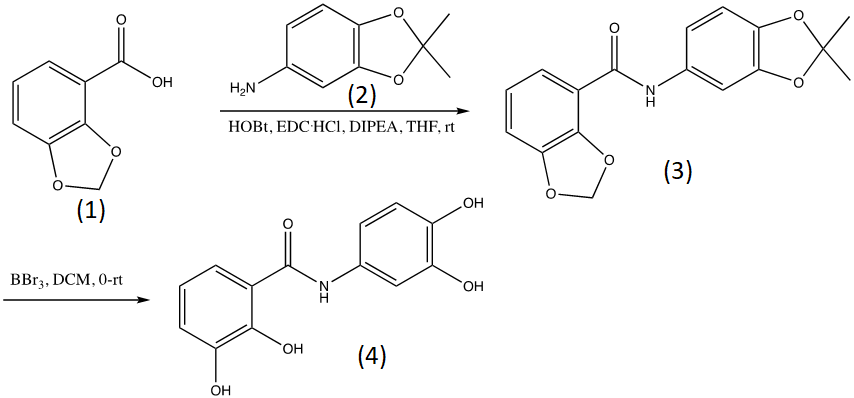


**S4 Fig.** **Chemical synthesis of Exebryl-1**. A brief description of the synthesis is as follows: to a suspension of protected benzoic acid **(1)** (0.64 g, 3.85 mmol) in THF (10.0 mL) was added hydorxybenzotriazole (HOBt, 0.52 g, 3.85 mmol), 1-ethyl-3-(3-dimethylaminopropyl)carbodiimide (EDC.HCl, 0.738 g, 3.85 mmol) and diisopropylethylamine (DIPEA, 1.83 mL, 10.5 mmol) at room temperature and stirred for 30 min. To this now clear solution the protected aniline **(2)** was added (0.48 g, 2.9 mmol) and the mixture was stirred at room temperature overnight. The solvent and excess reagent were removed under reduced pressure and the residue was purified on silica gel with 10% EtOAc/hexanes to provide 0.56 g (62%) of the intermediate **(3)** as pinkish solids. To a solution of the intermediate product **(3)** (0.56 g, 1.69 mmol) in DCM (17 mL) was added BBr_3_ (0.65 mL, 6.76 mmol) at 0^o^C, the mixture was allowed to warm to room temperature and stirred overnight. Methanol was added slowly until the suspension became clear. The solvents and excess reagent were removed under reduced pressure and the residue was purified on silica gel with 60% EtOAc/hexanes followed by 10% MeOH/DCM to provide the product with pink color. The material was suspended and stirred in DCM, filtered and washed with DCM to provide the final product as white solids (0.3 g, 68%).
