# Supplementary figures and images for: High-throughput screening of the ReFRAME, Pandemic Box, and COVID Box drug repurposing libraries against SARS-CoV-2 nsp15 endoribonuclease to identify small-molecule inhibitors of viral activity
Source: PLoS One. 2021 Apr 22;16(4):e0250019. doi: 10.1371/journal.pone.0250019 (PMC8062000; doi:10.1371/journal.pone.0250019)

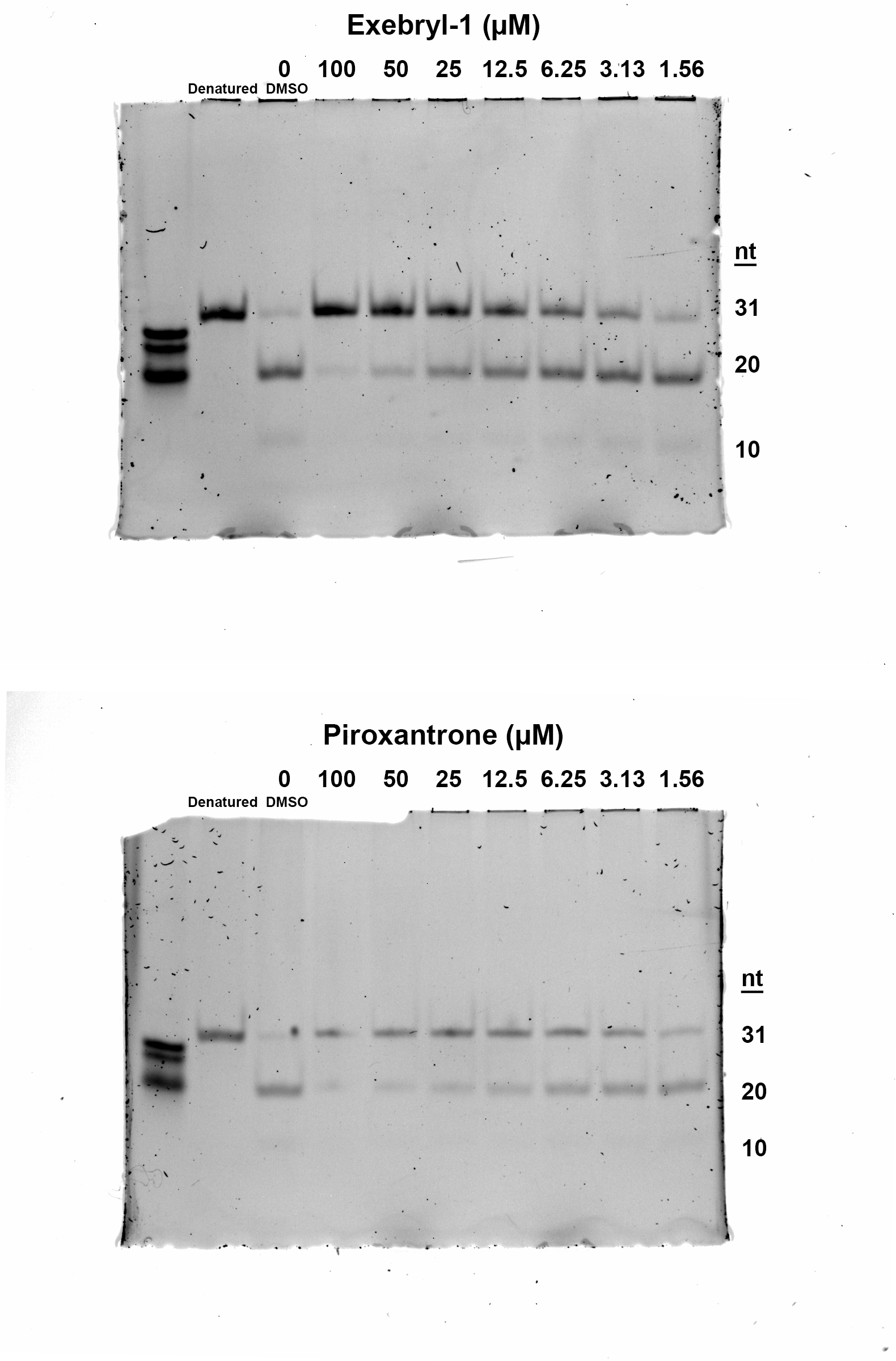

Supplement: S1 Raw images — (TIF) [file pone.0250019.s005.tif]
